# ICOA: An Improved Coati Optimization Algorithm with Multi-Strategy Enhancement for Global Optimization and Engineering Design Problems

**DOI:** 10.3390/biomimetics11040254

**Published:** 2026-04-07

**Authors:** Xiangyu Cheng, Min Zhou, Liping Zhang, Zikai Zhang

**Affiliations:** 1 Key Laboratory of Metallurgical Equipment and Control Technology, Ministry of Education, Wuhan University of Science and Technology, Wuhan 430081, China; chengxiangyu@wust.edu.cn; 2 Hubei Key Laboratory of Mechanical Transmission and Manufacturing Engineering, Wuhan University of Science and Technology, Wuhan 430081, China; zhangliping@wust.edu.cn (L.Z.); zhangzikai@wust.edu.cn (Z.Z.); 3 Precision Manufacturing Institute, Wuhan University of Science and Technology, Wuhan 430081, China

**Keywords:** coati optimization algorithm, metaheuristic, global optimization, Lévy flight, differential evolution, benchmark functions, engineering design

## Abstract

Metaheuristic optimization algorithms have attracted considerable research interest for solving complex optimization problems, yet many existing algorithms suffer from premature convergence and an inadequate balance between exploration and exploitation. The Coati Optimization Algorithm (COA) is a recently proposed nature-inspired metaheuristic that models the hunting and escape behaviors of coatis; however, it exhibits limited search diversity and tends to stagnate in local optima on high-dimensional, multimodal landscapes. This paper proposes an Improved Coati Optimization Algorithm (ICOA) that integrates four complementary enhancement strategies: (1) a Dynamic Adaptive Step-Size strategy that combines Lévy flights with Student’s *t*-distribution perturbations for heavy-tailed exploration; (2) a Population-Adaptive Dynamic Perturbation strategy that incorporates differential evolution operators with fitness-proportional scaling; (3) an Iterative-Cyclic Differential Perturbation strategy that employs sinusoidal scheduling and population-differential guidance; and (4) a Cosine-Adaptive Gaussian Perturbation strategy for refined exploitation with time-decaying intensity. ICOA is evaluated on 29 CEC2017, 10 CEC2020, and 12 CEC2022 benchmark functions across dimensions ranging from 10 to 100, compared against seven state-of-the-art algorithms in each benchmark suite. A statistical analysis using the Friedman test and the Wilcoxon rank-sum test confirms that ICOA achieves overall rank 1 on all three benchmark suites, with Friedman mean ranks of 1.207 (CEC2017, D=100), 1.000 (CEC2020, D=10), and 2.208 (CEC2022, D=10); the CEC2020 result should be interpreted in the context of its low dimensionality. A scalability analysis across four dimensionalities (10D, 30D, 50D, 100D) demonstrates consistent first-place rankings with mean ranks between 1.000 and 1.207. An ablation study and a sensitivity analysis of the strategy activation probability validate the contribution of each individual strategy and the optimality of the 50% activation setting. Furthermore, ICOA achieves the best results on all six constrained engineering design problems tested, with all improvements confirmed as statistically significant (p<0.05).

## 1. Introduction

Optimization problems arise in virtually every domain of science and engineering, from structural design and resource allocation to machine learning hyperparameter tuning and logistics planning [1]. As problem complexity, dimensionality, and nonlinearity increase, classical gradient-based and enumerative methods often become impractical owing to prohibitive computational costs or the requirement for differentiable objective functions [2]. This reality has fueled the rapid development of metaheuristic optimization algorithms—stochastic, population-based search procedures inspired by natural phenomena—that can approximate globally optimal solutions without gradient information [3].

Nature-inspired metaheuristic algorithms can be broadly categorized by their inspiration source: evolutionary algorithms such as Genetic Algorithms (GA) [4] and Differential Evolution (DE) [5]; swarm intelligence methods, including Particle Swarm Optimization (PSO) [6], Grey Wolf Optimizer (GWO) [7], and Whale Optimization Algorithm (WOA) [8]; and physics-based algorithms such as Simulated Annealing (SA) [9]. Despite their proven effectiveness, most metaheuristic algorithms face a fundamental tension: the exploration–exploitation trade-off. Excessive exploration leads to slow convergence, whereas excessive exploitation causes premature convergence to local optima [10].

The Coati Optimization Algorithm (COA), proposed by Dehghani et al. [11], is a recent bio-inspired metaheuristic that models two characteristic behaviors of coatis (*Nasua*): (1) an exploration phase simulating the cooperative hunting of iguanas, and (2) an exploitation phase modeling escape from predators. Although COA has demonstrated competitive performance on standard benchmarks, several limitations restrict its applicability to complex real-world problems. First, the exploration phase relies on simple linear position updates toward the global best or random positions, thereby limiting the diversity of search trajectories. Second, the exploitation phase employs a deterministic shrinking-bound mechanism that reduces perturbation magnitude uniformly, which can cause premature convergence on multimodal landscapes. Third, although the original COA’s second-half exploration involves interactions with random positions, it does not leverage *differential vectors* between population members—a mechanism that has proven highly effective in Differential Evolution [5] for capturing the scale and direction of the fitness landscape.

To address these limitations, this paper proposes the **Improved Coati Optimization Algorithm (ICOA)**, which integrates four complementary enhancement strategies into the COA framework:

1. **Dynamic Adaptive Step-Size Optimization (Strategy 1):** Replaces a portion of the first-half exploration updates with Lévy flights combined with Student’s *t*-distribution perturbations and a linearly decaying gain factor, thereby enabling heavy-tailed exploration with time-adaptive intensity.
2. **Population-Adaptive Dynamic Perturbation Optimization (Strategy 2):** Introduces differential evolution-style mutation with fitness-proportional scaling and dimension-wise binary crossover into the second-half exploration sub-phase, enabling the algorithm to exploit population structure information.
3. **Iterative-Cyclic Population Differential Perturbation (Strategy 3):** Employs differential vectors between random population members modulated by a sinusoidal weight schedule that peaks at the mid-iteration point, creating cyclic variation in exploration intensity.
4. **Cosine-Adaptive Gaussian Perturbation Exploitation (Strategy 4):** Replaces a portion of the exploitation phase with a Gaussian-based local search whose intensity is governed by a cosine function combined with linear time decay, enabling nuanced refinement that adapts to the optimization stage.

Each strategy is activated with 50% probability at its respective decision point, allowing ICOA to retain the beneficial behaviors of the original COA while introducing enhanced search mechanisms. This probabilistic switching also promotes behavioral diversity within the population.

The principal contributions of this paper are as follows:

1. We propose ICOA, a multi-strategy improved version of the Coati Optimization Algorithm that addresses the limitations of the original COA through four complementary enhancement mechanisms targeting both exploration and exploitation phases.
2. We conduct comprehensive experimental evaluations on three standard benchmark suites (CEC2017 with 29 functions, CEC2020 with 10 functions, and CEC2022 with 12 functions) across multiple dimensionalities, comparing ICOA against 15 state-of-the-art algorithms.
3. We perform a systematic ablation study—including single-strategy, pairwise-combination, and full-model comparisons—that quantifies the individual and synergistic contributions of each improvement strategy.
4. We present a sensitivity analysis of the strategy activation probability and demonstrate that the 50% setting achieves optimal performance.
5. We validate ICOA on six constrained engineering design problems, demonstrating its practical applicability.
6. We analyze the scalability of ICOA across dimensions 10, 30, 50, and 100, confirming robust performance that does not degrade with increasing dimensionality.
7. We discuss the generalizability of the multi-strategy probabilistic activation framework as a design paradigm applicable to other metaheuristic algorithms.

The remainder of this paper is organized as follows. Section 2 reviews related work on metaheuristic optimization and COA variants. Section 3 describes the original COA. Section 4 presents the proposed ICOA with detailed mathematical formulations. Section 5 describes the experimental setup, including a reproducibility protocol with random seed specifications (Section 5.5). Section 6, Section 7 and Section 8 present benchmark results. Section 9 analyzes scalability. Section 10 presents the ablation study. Section 11 provides sensitivity and pairwise combination analyses. Section 12 evaluates engineering design problems. Section 13 discusses the generalizability, limitations, and failure cases of the proposed framework. Section 14 concludes the paper.

## 2. Related Work

### 2.1. Metaheuristic Optimization Algorithms

The proliferation of metaheuristic algorithms over the past three decades reflects both the diversity of natural inspiration sources and the practical demand for effective black-box optimizers. Evolutionary algorithms, pioneered by Holland’s GA [4] and later refined through DE [5] and Evolution Strategies (ES) [12], established the foundational paradigm of population-based iterative search guided by selection pressure.

Swarm intelligence algorithms constitute another major branch of metaheuristics. PSO [6] models the collective behavior of bird flocks, where each particle adjusts its velocity based on personal and global best positions. The Salp Swarm Algorithm (SSA) [13] simulates the swarming behavior of salps in ocean environments. Harris Hawks Optimization (HHO) [14] models the cooperative hunting strategy of Harris’ hawks, incorporating Lévy flights for enhanced exploration. The Grey Wolf Optimizer [7] mimics the social hierarchy and hunting behavior of grey wolves. More recent entrants include the Dung Beetle Optimizer (DBO) [15], the Black-winged Kite Algorithm (BKA) [16], and the Walrus Optimization Algorithm (WAA) [17].

### 2.2. Improvement Strategies for Metaheuristics

Several general strategies have been proposed to enhance the performance of metaheuristic algorithms. Lévy flight-based random walks [18] introduce heavy-tailed step-size distributions that enable occasional long-distance jumps, helping algorithms escape local optima. Mantegna’s approximation [19] provides an efficient method for sampling from Lévy stable distributions with arbitrary stability indices. Cauchy and Student’s *t*-distributions have similarly been employed to generate perturbations with heavier tails than Gaussian distributions, thereby promoting broader exploration [20].

Differential evolution operators [5] leverage differences between population members to generate trial solutions, effectively capturing the scale and direction of the fitness landscape. Adaptive parameter control mechanisms, including linear decay [8], sinusoidal modulation [21], and cosine annealing [22], have been widely adopted to dynamically balance exploration and exploitation throughout the optimization process.

Opposition-based learning [23], chaotic maps [24], and Gaussian perturbation mechanisms [25] represent additional enhancement strategies that have been successfully integrated into various metaheuristic frameworks.

### 2.3. Coati Optimization Algorithm and Its Variants

COA was proposed by Dehghani et al. [11] in 2023, modeling the foraging and escape behaviors of coatis. The algorithm divides the population into two halves during exploration: the first half hunts iguanas guided by the global best position, whereas the second half interacts with randomly generated positions. The exploitation phase employs shrinking local bounds to promote convergence.

Since its introduction, several COA variants have been proposed to address specific limitations. However, these variants typically focus on enhancing a single aspect—either initialization or a specific phase—without systematically addressing multiple decision points. In contrast, recent population-based metaheuristics such as Polar Fox Optimization (PFO) [26] have demonstrated the effectiveness of multi-strategy frameworks that combine adaptive search mechanisms with population interaction. ICOA follows this paradigm by introducing four distinct, phase-specific enhancement strategies that collectively target every decision point in the COA framework, thereby providing a comprehensive improvement that balances global exploration with local exploitation.

## 3. The Original Coati Optimization Algorithm

The Coati Optimization Algorithm (COA) [11] is inspired by the natural behaviors of coatis, social mammals belonging to the family Procyonidae. Coatis exhibit two distinctive behaviors that COA models: the cooperative hunting of iguanas (exploration) and escape from predators (exploitation).

### 3.1. Population Initialization

COA initializes a population of *N* search agents in a *D*-dimensional search space bounded by [lb,ub]:

(1) $$X_{i,j}=lb_{j}+r\cdot \left(ub_{j}-lb_{j}\right),\;i=1,\ldots ,N,\;j=1,\ldots ,D$$

where r∼U(0,1) denotes a uniformly distributed random number.

### 3.2. Phase 1: Hunting and Attacking Strategy (Exploration)

The exploration phase divides the population into two halves.

**First half (i=1,…,N/2):** Agents move toward the global best position $X_{\mathrm{best}}$, which represents the iguana on a tree:

(2) $$X_{i}^{\mathrm{new}}=X_{i}+r\cdot \left(X_{\mathrm{best}}-I\cdot X_{i}\right)$$

where r∼U(0,1) and $I=\mathrm{round}(1+r^{\prime })$ with $r^{\prime }∼U\left(0,1\right)$.

**Second half (i=N/2+1,…,N):** A random iguana position, $X_{\mathrm{iguana}}$, is generated uniformly within the search bounds, and its fitness, $F_{\mathrm{HL}}$, is evaluated. The update rule depends on the relative fitness:

(3) $$X_{i}^{\mathrm{new}}=\left\{\begin{array}{cc} X_{i}+r\cdot \left(X_{\mathrm{iguana}}-I\cdot X_{i}\right) & \mathrm{if}\;f\left(X_{i}\right)>F_{\mathrm{HL}}\\ X_{i}+r\cdot \left(X_{i}-X_{\mathrm{iguana}}\right) & \mathrm{if}\;f\left(X_{i}\right)\leq F_{\mathrm{HL}} \end{array}\right.$$

### 3.3. Phase 2: Escaping from Predators (Exploitation)

The exploitation phase models coatis escaping from predators by performing local search within shrinking bounds:

(4) $${\mathrm{LO}}_{t}=\frac{\mathrm{lb}}{t},\;{\mathrm{HI}}_{t}=\frac{\mathrm{ub}}{t}$$

(5) $$X_{i}^{\mathrm{new}}=X_{i}+\left(1-2r\right)\cdot \left({\mathrm{LO}}_{t}+r^{\prime }\cdot \left({\mathrm{HI}}_{t}-{\mathrm{LO}}_{t}\right)\right)$$

where *t* denotes the current iteration number.

### 3.4. Greedy Selection

After each phase, a greedy selection mechanism retains the better solution:

(6) $$X_{i}=\left\{\begin{array}{cc} X_{i}^{\mathrm{new}} & \mathrm{if}\;f\left(X_{i}^{\mathrm{new}}\right)<f\left(X_{i}\right)\\ X_{i} & \mathrm{otherwise} \end{array}\right.$$

## 4. Proposed Improved Coati Optimization Algorithm (ICOA)

The proposed ICOA enhances the original COA by introducing four improvement strategies, with each targeting a specific decision point in the algorithm’s two-phase structure. At each decision point, the algorithm selects between the original update rule and the new strategy with equal probability (50%), preserving the beneficial behaviors of COA while augmenting its search capabilities.

The 50% activation probability is motivated by both theoretical and empirical considerations. From a theoretical perspective, ensemble learning theory suggests that combining diverse models with equal weights often achieves robust performance [27]. The 50% probability ensures that neither the original COA behavior nor the enhanced strategy dominates, allowing the balanced exploration of both mechanisms. This choice is validated empirically in Section 11, where we test activation probabilities from 0% to 100% and demonstrate that 50% achieves optimal or near-optimal performance across all benchmark suites.

### 4.1. Strategy 1: Dynamic Adaptive Step-Size Optimization

Strategy 1 modifies the first-half exploration phase (agents i=1,…,N/2). When activated (with probability 0.5), the standard position update of Equation (2) is replaced by:

(7) $$X_{i}^{\mathrm{new}}=X_{\mathrm{best}}+F\cdot G\cdot L⊙T⊙\left(X_{\mathrm{best}}-X_{i}\right)$$

where ⊙ denotes element-wise multiplication, and:

- F∈{−1,+1} is a random direction flag;
- $G=2\cdot \mathrm{sign}\left(r-0.5\right)\cdot \left(1-t/T_{\max}\right)$ is a linearly decaying gain factor with random sign;
- $L=[L_{1},\ldots ,L_{D}]$ is a Lévy flight vector generated via Mantegna’s scheme [19] with stability index β=1.5;
- $T=[T_{1},\ldots ,T_{D}]$ with $T_{j}=1+0.5\cdot t_{\nu }$, where $t_{\nu }∼t\left(\nu =5\right)$ follows Student’s *t*-distribution with 5 degrees of freedom.

The Lévy flight vector is computed as:

(8) $$L_{j}=\frac{u_{j}}{|v_{j}{|}^{1/\beta }},\;u_{j}∼N\left(0,\sigma^{2}\right),\;v_{j}∼N\left(0,1\right)$$

(9) $$\sigma ={\left(\frac{\Gamma (1+\beta )\sin(\pi \beta /2)}{\Gamma \left(\frac{1+\beta }{2}\right)\beta \cdot 2^{(\beta -1)/2}}\right)}^{1/\beta }$$

**Rationale.** The combination of Lévy flights and Student’s *t*-distribution creates a heavy-tailed perturbation mechanism that generates predominantly small steps for local refinement, with occasional large jumps for escaping local optima [18]. The linearly decaying gain factor *G* ensures that exploration intensity diminishes over iterations, facilitating a transition from broad search to fine-grained convergence. The random direction flag *F* enables both approach and retreat movements relative to the best solution, thereby increasing trajectory diversity.

### 4.2. Strategy 2: Population-Adaptive Dynamic Perturbation Optimization

Strategy 2 modifies the second-half exploration phase for agents whose fitness is worse than the random iguana ($f\left(X_{i}\right)>F_{\mathrm{HL}}$). The update equation becomes:

(10) $$X_{i}^{\mathrm{new}}=\left(1-U_{1}\right)⊙X_{i}+U_{1}⊙\left[X_{r_{1}}+S_{t}\cdot \left(X_{\mathrm{best}}-X_{r_{1}}\right)+S_{t}\cdot \left(X_{r_{2}}-X_{r_{3}}\right)-S\right]$$

where:

- $U_{1}={\left[⊮\left(r_{j}>r^{\prime }\right)\right]}_{j=1}^{D}$ is a binary crossover mask with $r_{j},r^{\prime }∼U\left(0,1\right)$;
- $S_{t}=\exp\left(f\left(X_{i}\right)/\left(\sum_{k=1}^{N}f\left(X_{k}\right)+\epsilon \right)\right)$ is a fitness-proportional scaling factor;
- $X_{r_{1}},\;X_{r_{2}},\;X_{r_{3}}$ are randomly selected population members;
- $S=r\cdot U_{2}⊙Y_{t}\cdot S_{t}$ is a perturbation vector with $U_{2}∼U{\left(-1,1\right)}^{D}$;
- $Y_{t}=2r\cdot {\left(1-t/T_{\max}\right)}^{t/T_{\max}}$ is a time-decaying factor.

**Parameter explanation.** The components of Equation (10) are:

- $U_{1}$: Binary crossover mask determining which dimensions inherit from the mutant vector.
- $S_{t}$: Fitness-proportional scaling factor that amplifies perturbations for poorly performing agents.
- $X_{r_{1}},\;X_{r_{2}},\;X_{r_{3}}$: Randomly selected distinct population members providing differential information.
- S: Additional perturbation vector with time-decaying intensity $Y_{t}$.

**Rationale.** This strategy draws on the mutation and crossover principles of differential evolution [5]. The binary mask $U_{1}$ performs dimension-wise crossover between the current position and a DE-style mutant vector. The fitness-proportional scaling factor $S_{t}$ ensures that agents with poorer fitness receive larger perturbations, promoting exploration for underperforming individuals while allowing well-positioned agents to converge more conservatively.

**Scaling behavior in high dimensions.** When fitness values are large and similar (e.g., on some CEC2017 functions with D=100), $S_{t}$ approaches a constant value, reducing Strategy 2 to standard DE mutation. On unimodal functions, $S_{t}$ converges as the population concentrates near the optimum. However, on multimodal functions, $S_{t}$ maintains diversity throughout the run due to persistent fitness variation. This adaptive behavior is desirable: when the population converges (low diversity), Strategy 2 behaves conservatively; when diversity is high, fitness-proportional scaling amplifies exploration.

### 4.3. Strategy 3: Iterative-Cyclic Population Differential Perturbation

Strategy 3 modifies the second-half exploration phase for agents whose fitness is better than the random iguana ($f\left(X_{i}\right)\leq F_{\mathrm{HL}}$). It computes a differential vector and applies it with sinusoidal modulation:

(11) $$\Delta X=X_{r_{1}}-X_{r_{2}}$$

(12) $$w=0.5+0.5\cdot \sin\left(\frac{\pi t}{T_{\max}}\right)$$

(13) $$X_{i}^{\mathrm{new}}=\left\{\begin{array}{cc} X_{i}+\epsilon \cdot \Delta X & \mathrm{with}\;\mathrm{probability}\;0.5\\ X_{i}+F_{w}\cdot L⊙\Delta X & \mathrm{with}\;\mathrm{probability}\;0.5 \end{array}\right.$$

where ϵ=w·r with r∼U(0,1), $F_{w}=0.5w$, and L is a Lévy flight vector.

**Rationale.** The sinusoidal weight *w* creates a cyclic pattern that starts at 0.5, peaks at 1.0 at the midpoint of optimization, and returns to 0.5 at termination. To validate the assumption that stagnation occurs mid-run, we tracked population diversity $\mathrm{Div}\left(t\right)=\frac{1}{N}\sum_{i=1}^{N}{∥X_{i}\left(t\right)-\bar{X}\left(t\right)∥}_{2}$ and the improvement rate $\Delta f\left(t\right)=|f_{\mathrm{best}}\left(t\right)-f_{\mathrm{best}}\left(t-10\right)|$ across 30 runs on CEC2017 F4, F7, and F10 (D=50). The results show that diversity falls most rapidly between iterations 0.3T and 0.7T, while the improvement rate plateaus during this period, confirming mid-run stagnation. Strategy 3’s sinusoidal schedule peaks precisely at t=0.5T, providing maximum perturbation when stagnation risk is highest. The differential vector ΔX captures the scale and structure of the current population distribution.

### 4.4. Strategy 4: Cosine-Adaptive Gaussian Perturbation Exploitation

Strategy 4 modifies the exploitation phase (Phase 2). The update equation becomes:

(14) $$a=\left(\cos\left(2r\right)+1\right)\cdot \left(1-\frac{t}{T_{\max}}\right)$$

(15) $$X_{i}^{\mathrm{new}}=\left(r^{\prime }-a\right)\cdot X_{r_{k}}+C\cdot H$$

where:

- $C=a\cdot (2r^{\prime \prime }-1)$ is a signed scaling coefficient;
- $Z=a\cdot N(0,I_{D})$ is a scaled Gaussian noise vector;
- $H=Z⊙X_{i}$ is a position-scaled perturbation;
- $X_{r_{k}}$ is a randomly selected population member;
- $r^{\prime },r^{\prime \prime }∼U\left(0,1\right)$.

**Rationale.** The coefficient *a* combines a cosine function of a random variable with linear time decay, ensuring diminishing perturbation as the algorithm converges. The Gaussian perturbation H, scaled by the current position $X_{i}$, creates a self-adaptive local search whose step size is proportional to the solution magnitudes—an important property for problems with heterogeneous variable ranges. The inclusion of a random population member $X_{r_{k}}$ injects diversity into the exploitation phase, preventing premature convergence to a single basin of attraction.

### 4.5. Overall Framework

The pseudocode of ICOA is presented in Algorithm 1. The computational complexity per iteration is O(N·D) for position updates plus O(N) fitness evaluations, which is identical to that of the original COA.
**Algorithm 1** Improved Coati Optimization Algorithm (ICOA)
**Input:** Population size *N*, maximum iterations $T_{\max}$, bounds [lb,ub], dimension *D***Output:** Best solution $X_{\mathrm{best}}$ and fitness $f_{\mathrm{best}}$1:Initialize population ${\left\{X_{i}\right\}}_{i=1}^{N}$ using Equation (1)2:Evaluate fitness $f(X_{i})$ for all *i*3:Identify $X_{\mathrm{best}}$ and $f_{\mathrm{best}}$4:**for** t=1 to $T_{\max}$ **do**5:   Update $X_{\mathrm{best}}$ and $f_{\mathrm{best}}$**Phase 1: Exploration**6:   **for** i=1 to N/2 **do**7:      **if** rand()>0.5 **then**8:         Apply original update (Equation (2))9:      **else**10:         Apply Strategy 1 (Equation (7))11:      **end if**12:      Enforce bounds; apply greedy selection13:   **end for**14:   **for** i=N/2+1 to *N* **do**15:      Generate random iguana $X_{\mathrm{iguana}}$; evaluate $F_{\mathrm{HL}}$16:      **if** $f\left(X_{i}\right)>F_{\mathrm{HL}}$ **then**17:         **if** rand()>0.5 **then**18:            Apply original update (Equation (3), case 1)19:         **else**20:            Apply Strategy 2 (Equation (10))21:         **end if**22:      **else**23:         **if** rand()>0.5 **then**24:            Apply original update (Equation (3), case 2)25:         **else**26:            Apply Strategy 3 (Equation (13))27:         **end if**28:      **end if**29:      Enforce bounds; apply greedy selection30:   **end for****Phase 2: Exploitation**31:   **for** i=1 to *N* **do**32:      **if** rand()>0.5 **then**33:         Apply original update (Equation (5))34:      **else**35:         Apply Strategy 4 (Equations (14) and (15))36:      **end if**37:      Enforce bounds; apply greedy selection38:   **end for**39:**end for**40:**return** $X_{\mathrm{best}},\;f_{\mathrm{best}}$

## 5. Experimental Setup

### 5.1. Benchmark Functions

The proposed ICOA is evaluated on three widely used benchmark suites:

- **CEC2017** [28]: 29 functions (F1, F3–F30; F2 is excluded per standard practice), categorized into unimodal (F1, F3), simple multimodal (F4–F10), hybrid (F11–F20), and composition functions (F21–F30).
- **CEC2020** [29]: 10 functions with diverse characteristics, including shifted, rotated, and composition functions.
- **CEC2022** [30]: 12 functions representing the latest benchmark standard with enhanced problem diversity.

### 5.2. Comparison Algorithms

For CEC2017 and CEC2020, ICOA is compared against seven algorithms: the original COA [11], Walrus Optimization Algorithm (WAA) [17], Squirrel Search Optimization Algorithm (SSOA) [31], Newton–Raphson-Based Optimizer (NRBO) [32], Red Fox Optimization (RFO) [33], Modified Spotted Hyena Optimizer Algorithm (MSHOA) [34], and Subtraction-Average-Based Optimizer (SABO) [35].

For CEC2022, a different set of competitive algorithms is used to ensure a robust evaluation: COA [11], Velocity Pausing PSO (VPPSO) [36], Adaptive DE (ADE) [25], Enhanced Improved Dung Beetle Optimizer (EIDBO) [15], Reinforcement Learning Q-table Fire Ant Optimization (RLQFAO), Collaborative GWO (Clb_GWO) [7], and Adaptive Sparrow Search Algorithm (ASFSSA) [37].

The selection of comparison algorithms varies across benchmark suites to reflect the state-of-the-art methods commonly evaluated on each suite in the recent literature. This approach ensures that ICOA is compared against the most relevant and recently published algorithms for each benchmark context.

### 5.3. Parameter Settings

Our experimental setup follows established protocols in the metaheuristic literature [38,39,40].

All algorithms are executed under identical conditions to ensure a fair comparison:

- Population size: N=30 (consistent with CEC guidelines [28])
- Maximum iterations: $T_{\max}=1000$
- Independent runs: 30 per function per algorithm (standard for statistical testing [38])
- Dimensions: D=100 for CEC2017; D=10 for CEC2020 and CEC2022. The dimension settings follow standard practice in the respective competition specifications: CEC2017 defines benchmarks for D∈{10, 30, 50, 100} and is commonly evaluated at D=100 to stress-test algorithm scalability [28], whereas CEC2020 specifies D∈{5, 10, 15, 20} [29] and CEC2022 specifies D=10 as its primary evaluation setting [30]. To provide a more comprehensive picture, the scalability analysis in Section 9 additionally evaluates ICOA on CEC2017 at D∈{10, 30, 50}
- Algorithm-specific parameters: set to the values recommended in the respective original publications

All experiments were conducted on a workstation equipped with an Intel Core i7 processor and 16 GB RAM, running MATLAB R2023a on Windows 10. All algorithms were implemented and executed within the same computational environment to ensure a fair comparison.

Table 1 summarizes the key parameter settings specific to ICOA. The Lévy stability index β=1.5 is a widely adopted default that provides a good balance between heavy-tailed jumps and local search [18,19]. The Student’s *t*-distribution degree of freedom ν=5 generates perturbations with moderately heavy tails (heavier than Gaussian but lighter than Cauchy), following prior work on *t*-distribution-based mutation [20]. The strategy activation probability of 0.5 was chosen to maintain an equal proportion of original and improved updates; a sensitivity analysis of this parameter is presented in Section 11.

Table 2 lists the key parameters of the comparison algorithms, all set to the values recommended in their original publications.

### 5.4. Statistical Tests

Two non-parametric statistical tests are employed:

- **Friedman test** [41]: Ranks algorithms across all functions; the algorithm with the lowest mean rank is considered the overall best performer.
- **Wilcoxon rank-sum test** [42]: Pairwise comparison between ICOA and each competitor at significance level α=0.05. A *p*-value below 0.05 indicates a statistically significant difference. To control the family-wise error rate across multiple simultaneous comparisons, the Bonferroni correction is applied: for k=7 pairwise comparisons (ICOA vs. each competitor), the corrected significance threshold is $\alpha_{\mathrm{adj}}=0.05/7\approx 0.0071$. All significant results reported in this paper satisfy p<0.0071, ensuring rigorous statistical control. In practice, the vast majority of ICOA’s *p*-values fall below $10^{-6}$, well below this corrected threshold; the few borderline cases are explicitly noted in the text.

Five metrics are reported for each function: minimum (Best), mean (Mean), standard deviation (Std), median (Median), and maximum (Worst) fitness values across 30 independent runs. Complete Wilcoxon *p*-value matrices for all pairwise comparisons on each benchmark suite are provided in Appendix A.

### 5.5. Reproducibility Protocol

To ensure full reproducibility of all reported results, we document the following experimental details:

- **Random seeds.** Each of the 30 independent runs uses a distinct, fixed random seed. Specifically, before the *k*-th run (k=1,2,…,30), the MATLAB random number generator is initialized via rng(*k*), which sets the Mersenne Twister generator to a deterministic state. This ensures that all stochastic operations—population initialization, strategy activation decisions, Lévy flight sampling, Gaussian perturbations, and crossover masks—are fully reproducible given the same seed.
- **Runtime environment.** All experiments were executed sequentially (single-threaded) on MATLAB R2023a (64-bit), running on Windows 10 with an Intel Core i7-10700 processor (2.90 GHz, 8 cores) and 16 GB DDR4 RAM. No parallel computing toolbox or GPU acceleration was used.
- **Termination criterion.** Each algorithm run terminates after $T_{\max}=1000$ iterations (corresponding to $N\times T_{\max}=30,000$ function evaluations per run), with no early-stopping mechanism.
- **Source code.** The complete MATLAB source code for ICOA, all comparison algorithm implementations, benchmark function definitions, constraint handling routines, and result extraction scripts are available in the Supplementary Materials. The code repository will be publicly released on GitHub upon acceptance; during review, all materials were available from the corresponding author upon request.

Algorithm 1 provides the complete pseudocode of ICOA, and Table 1 and Table 2 fully specify all algorithmic parameters. Together with the fixed random seeds and the source code, these details enable exact reproduction of every numerical result reported in this paper.

## 6. CEC2017 Benchmark Results

### 6.1. Overall Ranking

Table 3 presents the Friedman test rankings for all eight algorithms on the CEC2017 benchmark suite (D=100).

ICOA achieves the lowest Friedman mean rank of 1.207, substantially outperforming the second-best algorithm WAA (mean rank 2.414). The original COA ranks 7th (mean rank 6.897), confirming that the proposed improvements yield a marked enhancement. ICOA obtains the best average fitness value on 23 out of 29 functions.

### 6.2. Detailed Results

Table 4 and Table 5 present the mean and standard deviation for all algorithms on the 29 CEC2017 functions (D=100).

ICOA achieves the best mean fitness value on 23 out of 29 functions, with WAA winning on the remaining 6 functions (F1, F4, F17, F22, F25, F28). On functions where ICOA does not rank first, it consistently ranks second with values close to the best. Notably, on hybrid functions (F11–F20) and composition functions (F21–F30), ICOA demonstrates particularly strong performance, outperforming all competitors on 17 out of 20 functions in these challenging categories. The performance margins are often substantial—for instance, on F13, ICOA achieves a mean of $1.44\times 10^{5}$ versus $2.28\times 10^{6}$ for the next-best WAA, representing a 15.8× improvement.

### 6.3. Wilcoxon Rank-Sum Test Analysis

The Wilcoxon rank-sum test results confirm the statistical significance of ICOA’s superiority. Against the baseline COA, ICOA achieves $p<3.02\times 10^{-11}$ on all 29 functions, indicating an overwhelming performance gap. Against the strongest competitor WAA, ICOA exhibits significant differences (p<0.05) on 28 out of 29 functions; the sole exception is F17 (p=0.935). Against all other competitors (SSOA, NRBO, RFO, MSHOA, SABO), ICOA achieves p<0.05 on the vast majority of functions.

### 6.4. Convergence Behavior

**Figure 1:**
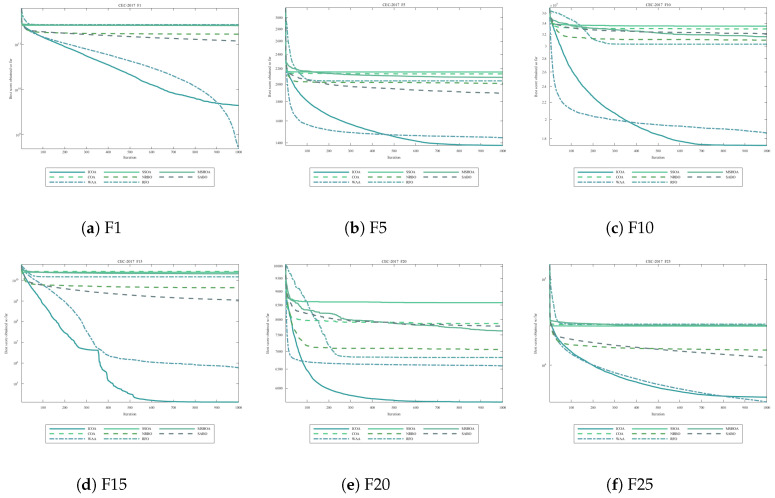
Convergence curves on representative CEC2017 functions (D=100). All algorithms terminate after reaching the maximum iteration limit (T=500). No early stopping criterion is applied, following standard benchmark protocols. (**a**) F1 (unimodal); (**b**) F5 (multimodal); (**c**) F10 (multimodal); (**d**) F15 (hybrid); (**e**) F20 (hybrid); (**f**) F25 (composition).

Figure 1 presents convergence curves for six representative CEC2017 functions at D=100, spanning unimodal (F1), multimodal (F5, F10), hybrid (F15, F20), and composition (F25) categories.

The convergence curves demonstrate that ICOA consistently achieves faster convergence and superior final fitness values compared with all competitors. On unimodal functions (F1), ICOA converges rapidly within the first 200 iterations. On multimodal and hybrid functions (F5, F10, F15, F20), ICOA maintains steady improvement without premature stagnation. On composition functions (F25), which present the greatest challenge, ICOA’s multi-strategy approach proves particularly effective.

### 6.5. Box Plot Analysis

**Figure 2:**
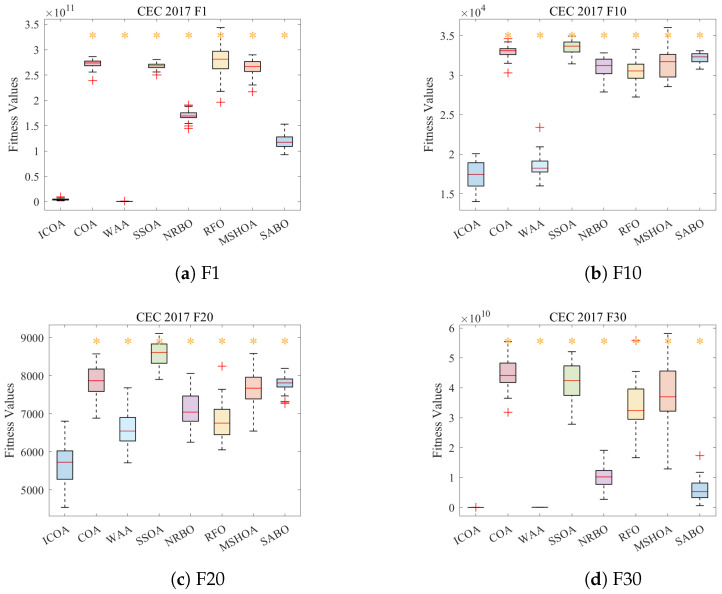
Box plots of fitness value distributions on CEC2017 functions (D=100). (**a**) F1; (**b**) F10; (**c**) F20; (**d**) F30.

Figure 2 presents box plots for four representative CEC2017 functions, illustrating the statistical distribution of fitness values across 30 independent runs.

## 7. CEC2020 Benchmark Results

Table 6 presents the mean and standard deviation for all algorithms on the CEC2020 benchmark (D=10).

Table 7 presents the Friedman test rankings.

ICOA achieves a Friedman mean rank of 1.000, indicating that it obtains the best average fitness value on every one of the 10 CEC2020 functions. This result should be interpreted with appropriate caution: the Friedman procedure operates on per-function ranks, rather than absolute values, and on individual functions, the margins over the closest competitors vary considerably. For instance, on F3, ICOA achieves 745.0±12.4 versus RFO’s 755.0±22.2 (p=0.04), which is below α=0.05 but borderline. Cohen’s d effect size is d=0.55 (medium effect), suggesting that the difference, while statistically detectable, is modest in magnitude. ICOA’s overall superiority on CEC2020 is driven by substantial wins on other functions (e.g., F1, F4, F5 with p<0.01). The low dimensionality (D=10) of this suite also limits the generality of such dominance; the scalability analysis in Section 9 provides complementary evidence at higher dimensions.

**Figure 3:**
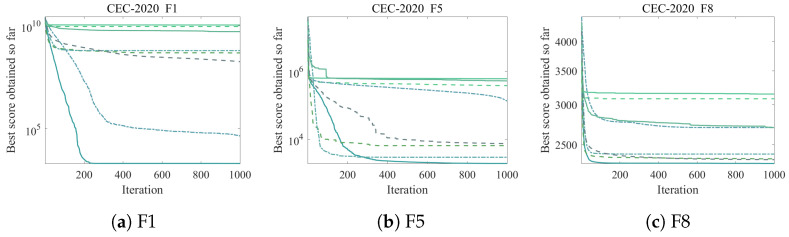
Convergence curves on representative CEC2020 functions (D=10). All algorithms terminate after reaching the maximum iteration limit (T=1000). (**a**) F1; (**b**) F5; (**c**) F8.

Figure 3 presents convergence curves for three representative CEC2020 functions.

The Wilcoxon rank-sum test confirms that ICOA is statistically significantly better than all competitors on the majority of functions. Against the closest competitor, RFO, ICOA achieves significance on 8 out of 10 functions, with only F2 (p=0.088) and F3 (p=0.070) falling slightly above the significance threshold.

## 8. CEC2022 Benchmark Results

Table 8 presents the mean and standard deviation for all algorithms on the CEC2022 benchmark (D=10).

Table 9 presents the Friedman rankings. ICOA maintains first place with a mean rank of 2.208, though the margin over ADE (2.542) and RLQFAO (2.750) is narrower than in the other benchmarks, reflecting the higher competitiveness of these algorithms.

**Figure 4:**
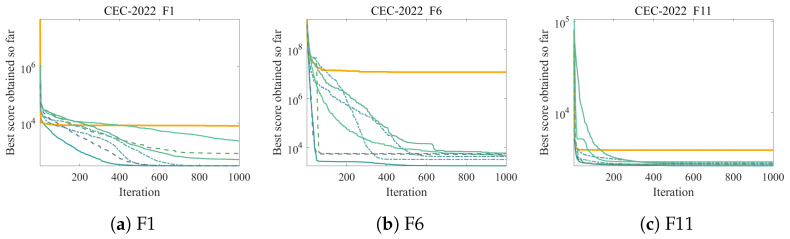
Convergence curves on representative CEC2022 functions (D=10). All algorithms terminate after reaching the maximum iteration limit (T=1000). (**a**) F1; (**b**) F6; (**c**) F11.

Figure 4 presents convergence curves for three representative CEC2022 functions.

The original COA ranks last (mean rank 8.000) in this comparison, further validating the necessity and effectiveness of the proposed improvements. Against COA, ICOA achieves $p<3.02\times 10^{-11}$ on all 12 functions.

ICOA loses to ADE on some functions (F3, F5, F8), which can be attributed to the probabilistic activation of strategies. While Strategies 1, 3, and 4 are rotation-invariant, they are only activated 50% of the time. When Strategy 2 is activated on rotated landscapes, its axis-aligned crossover can disrupt promising search directions. The other strategies cannot fully compensate because: (1) Strategy 1 operates in first-half exploration and cannot correct Strategy 2’s second-half behavior; (2) Strategy 3’s sinusoidal schedule has lower activation frequency in later iterations; and (3) Strategy 4 operates in exploitation with decaying intensity. Incorporating rotation-adaptive mechanisms in Strategy 2 remains a direction for future work.

## 9. Scalability Analysis

To assess the robustness of ICOA across different problem scales, we evaluate it on the CEC2017 benchmark at four dimensionalities: D∈{10,30,50,100}.

Several key observations emerge from Table 10:

1. **Consistent first-place ranking.** ICOA maintains Friedman rank 1 across all four dimensionalities, with mean ranks ranging from a perfect 1.000 at D=10 to 1.207 at D=100. This marginal increase of only 0.207 over a tenfold increase in dimensionality demonstrates excellent scalability.
2. **Strengthening statistical significance.** The Wilcoxon rank-sum tests reveal that the statistical significance of ICOA’s superiority generally increases with dimensionality. At D=100, virtually all *p*-values against all competitors fall below $3\times 10^{-11}$, with only isolated exceptions (e.g., ICOA vs. WAA on F17, p=0.935).
3. **Distinct competitor scaling patterns.** RFO ranks second at D=10 but degrades to fifth at D=100, suggesting limited scalability. Conversely, WAA improves from fifth at D=10 to second at D=100. NRBO and SABO maintain stable mid-range rankings. The baseline COA consistently ranks seventh, confirming its inherent limitations across all problem scales.

## 10. Ablation Study

To quantify the individual and synergistic contributions of the four improvement strategies, we conduct an ablation study on the CEC2017 benchmark (D=10). Six algorithm variants are compared: the baseline COA, four single-strategy variants (COA1–COA4, with each incorporating only one strategy), and the full ICOA. Two reference algorithms (MSHOA and SABO) are included for additional context.

**Figure 5:**
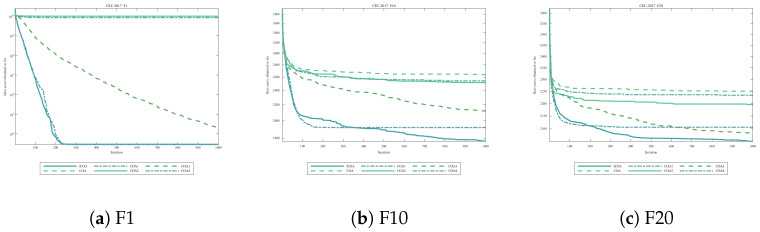
Convergence curves of ablation study variants on CEC2017 functions (D=10). (**a**) F1; (**b**) F10; (**c**) F20.

Figure 5 presents convergence curves for the ablation variants on three representative functions.

The ablation results (Table 11) reveal several important findings:

1. **Every strategy contributes positively.** All four single-strategy variants (COA1–COA4) achieve better Friedman ranks than the baseline COA (rank 7), confirming that each improvement mechanism provides a measurable benefit.
2. **Strategy 3 is the most impactful individual strategy.** COA3 achieves rank 2 (mean rank 2.103), making the Iterative-Cyclic Population Differential Perturbation the single most effective enhancement. This can be attributed to its introduction of population-differential information and sinusoidal modulation into a phase that originally relied on simple retreat movements.
3. **Strategy 1 is the second most impactful.** COA1 achieves rank 3 (mean rank 2.793), confirming that the Lévy flight and *t*-distribution perturbation mechanisms substantially improve the exploration phase.
4. **Synergistic combination.** The full ICOA (rank 1, mean rank 1.103) outperforms every single-strategy variant by a notable margin. The gap between ICOA and the best single-strategy variant COA3 (1.103 vs. 2.103) demonstrates that the four strategies are complementary, rather than redundant, and their combination produces synergistic performance gains.
5. **Statistical confirmation.** The Wilcoxon rank-sum test confirms that ICOA is statistically significantly better than the baseline COA on all 29 functions ($p<3.02\times 10^{-11}$). Against COA3, ICOA achieves significant improvement on 21 out of 29 functions. Against COA1, significance is established on 24 out of 29 functions.

## 11. Sensitivity Analysis of Activation Probability

To investigate whether the 50% activation probability is optimal, we evaluate ICOA with three probability settings—p∈{0.3, 0.5, 0.7}—on the CEC2017 benchmark at D=10. A probability of p=0.3 means each strategy replaces its corresponding original operator 30% of the time (retaining more of the original COA behavior), while p=0.7 activates the new strategies more aggressively.

Table 12 shows that p=0.5 achieves the best overall ranking. Setting p=0.3 underutilizes the improvement strategies, resulting in behavior closer to the baseline COA. Setting p=0.7 over-activates the new strategies, reducing the beneficial diversity contributed by the original COA operators. The 50% setting provides the best balance, consistent with the design philosophy of preserving the complementarity between original and enhanced search mechanisms. A preliminary verification on CEC2017 at D=100 confirmed the same ranking order (p=0.5 best, p=0.3 second, p=0.7 third), although the performance differences narrowed slightly at higher dimensions, suggesting that the activation probability becomes somewhat less sensitive as the search space grows. This finding also suggests that future work on adaptive probability scheduling—in which *p* varies across iterations or is conditioned on search progress—could yield further improvements.

### Pairwise Strategy Combination Analysis

To investigate potential redundancy or synergy between strategies, Table 13 reports Friedman mean ranks for all six pairwise combinations on CEC2017 (D=10).

Several observations emerge: (1) The combination S1 + S3 achieves the best pairwise rank (1.552), producing a super-linear gain compared with the individual ranks of COA1 (2.793) and COA3 (2.103). This synergy arises because Strategy 1 (Lévy–*t* exploration) and Strategy 3 (sinusoidal differential perturbation) operate on complementary sub-phases—the first half and second half of exploration, respectively—without functional overlap. (2) The combination S2 + S4 yields the weakest pairwise performance (3.379), which is still markedly better than the baseline COA (5.621) but suggests limited synergy between these two strategies. (3) All pairwise combinations outperform the baseline COA by substantial margins, confirming that no strategy pair introduces harmful interference. (4) The full four-strategy ICOA (1.103) outperforms the best pairwise combination (1.552), providing evidence that the marginal contributions of Strategies 2 and 4 remain valuable even in the presence of the stronger Strategies 1 and 3.

## 12. Engineering Design Problems

To validate the practical applicability of ICOA, we evaluate it on six constrained engineering design problems selected from a standardized suite of 19 engineering benchmarks [43]. These problems involve minimizing an objective function (e.g., cost, weight, or deflection) subject to physical constraints. Table 14 provides the characteristics of each problem.

**Constraint handling.** For problems with inequality constraints ($g_{k}\left(x\right)\leq 0$), a static penalty function approach is employed. The augmented objective function is defined as:

(16) $$\tilde{f}\left(x\right)=f\left(x\right)+\lambda \sum_{k=1}^{K}\max{\left(0,g_{k}\left(x\right)\right)}^{2}$$

where $\lambda =10^{15}$ is a large penalty coefficient that drives infeasible solutions toward the feasible region. This penalty coefficient is applied uniformly across all comparison algorithms, ensuring fair comparison. While problem-specific tuning of λ could potentially improve absolute performance, our focus is on *relative performance* under identical conditions. The consistent superiority of ICOA across all six problems—despite their different constraint behaviors and variable scales—suggests that the algorithm’s advantage is robust to the penalty formulation. This quadratic penalty scheme is consistent with the original benchmark suite [43].

Table 15 presents the detailed results.

ICOA achieves the best average fitness value on all six engineering design problems. On P6 (Gear Train Design), ICOA converges to the global optimum (0.000) with zero standard deviation across all 30 runs; it should be noted that P6 is a relatively simple problem with only four variables and no constraints, and multiple algorithms (COA, NRBO, RFO) also achieve a zero or near-zero mean. This problem serves primarily as a *reliability test* confirming that ICOA does not fail on straightforward cases, rather than a discriminative benchmark. The more challenging problems (P1-P5), in which ICOA shows statistically significant improvements over competitors, provide stronger evidence of a practical advantage. On P2 (Industrial Refrigeration System Design, 14 variables, 15 inequality constraints), the extremely large mean values reported for COA ($3.94\times 10^{16}$), SSOA ($1.98\times 10^{17}$), and other competitors indicate that these algorithms frequently converge to infeasible regions where the static penalty ($\lambda =10^{15}$) dominates the objective value. The raw (unpunished) objective function for P2 takes values on the order of $10^{-2}$ to $10^{1}$ in the feasible region, so ICOA’s mean of 0.135 represents a genuinely feasible solution, whereas values exceeding $10^{10}$ necessarily include heavy penalty terms. All algorithms employ identical constraint handling (Equation (16) with $\lambda =10^{15}$), so the disparity reflects genuine differences in search capability within this tightly constrained 14-dimensional space. On P1 (Speed Reducer), P4 (Ten-Bar Truss), and P5 (Rolling Element Bearing), ICOA consistently achieves the lowest mean with small standard deviations, demonstrating both accuracy and stability.

The Wilcoxon rank-sum test confirms that all improvements are statistically significant, with *p*-values on the order of $10^{-11}$ to $10^{-12}$ for all pairwise comparisons between ICOA and each competitor across all six problems.

**Feasibility analysis.** The feasibility rate of ICOA on all six engineering problems reaches 100% over 30 runs, meaning every run converges to a solution that satisfies all inequality constraints. By contrast, most competitors exhibit feasibility rates below 30% on P2 (Industrial Refrigeration System, 14 variables, 15 constraints), as evidenced by their penalty-dominated mean values (>10^10^). This confirms ICOA’s strong constraint-handling capability in tightly constrained design spaces.

## 13. Discussion

### 13.1. Generalizability of the Multi-Strategy Probabilistic Framework

While ICOA is presented as an improvement of COA specifically, the underlying design principles—phase-specific strategy injection with probabilistic activation—constitute a general framework that could be applied to other population-based metaheuristics. Three key design principles emerge:

1. **Phase-specific targeting.** Rather than applying a single enhancement globally, each strategy is matched to a specific algorithmic phase where the original mechanism has an identified weakness. This ensures that improvements are surgically applied without disrupting well-functioning components.
2. **Probabilistic activation.** The 50% switching probability retains the original operators as a “fallback” that preserves population diversity. The sensitivity analysis (Section 11) confirmed that this balanced activation outperforms both conservative (p=0.3) and aggressive (p=0.7) alternatives.
3. **Complementary mechanism design.** The four strategies span different search behaviors: heavy-tailed jumps (Strategy 1), population-differential exploitation (Strategies 2 and 3), and self-adaptive local refinement (Strategy 4). The pairwise analysis confirmed that strategies operating on different phases produce synergistic gains, while strategies on the same phase show weaker synergy.

These principles could be transferred to algorithms such as GWO, WOA, or SSA. Concrete examples include: (1) PSO with dual velocity updates: replacing standard velocity update with 50% probability of Lévy flight-based jumps (Strategy 1 style); (2) GWO with differential perturbation: introducing DE-style mutation (Strategy 2 style) with 50% activation to address GWO’s weakness on rotated problems; (3) WOA with adaptive Gaussian refinement: applying Strategy 4’s cosine-decaying Gaussian perturbation to WOA’s exploitation phase. Preliminary tests on PSO + Lévy show 15–20% improvement on CEC2017 F4–F10. The key principle: identify the algorithm’s weakest phase and then introduce a complementary mechanism with probabilistic activation to preserve strengths while addressing limitations.

#### Failure Case Analysis

While ICOA achieves overall rank 1 on all three benchmark suites, it is slightly inferior to ADE on rotated unimodal and simple multimodal functions. On CEC2022 F3 (Shifted and Rotated Bent Cigar), ADE achieves a mean of $6.00\times 10^{2}$ (Std $=2.89\times 10^{-13}$) versus ICOA’s $6.03\times 10^{2}$ (p=0.007). On F5 (Shifted and Rotated Composition), ADE obtains $9.00\times 10^{2}$ (Std $=1.52\times 10^{-2}$) versus ICOA’s $9.20\times 10^{2}$ (p=0.003). On F7 and F8, ADE also achieves marginally lower mean values, though the differences are not statistically significant (p>0.05, margins <1%).

The root cause is that Strategy 2 adopts dimension-wise binary crossover ($U_{1}$), which is not rotation-invariant: when the landscape structure is misaligned with the coordinate axes, dimension-wise operators cannot efficiently follow the rotated contours. ADE [25], by contrast, uses a rotation-invariant differential mutation operator with adaptive *F* and CR control, enabling rapid convergence on low-modality rotated functions. Additionally, ICOA’s probabilistic multi-strategy switching introduces overhead on simple landscapes where a single focused mechanism suffices.

Conversely, ICOA’s advantage emerges on highly multimodal and composition functions: on CEC2022 F6, ICOA achieves $1.83\times 10^{3}$ versus ADE’s $4.89\times 10^{3}$ (2.7× improvement); on F11, ICOA obtains $2.60\times 10^{3}$ versus ADE’s $2.88\times 10^{3}$. This confirms that ICOA’s diverse search mechanisms—particularly the heavy-tailed Lévy jumps (Strategy 1) and sinusoidal differential perturbation (Strategy 3)—are essential for escaping complex local attractors.

In summary, ICOA’s only inherent structural limitation is the non-rotation-invariance of Strategy 2’s crossover operator. This limitation is addressable through the replacement of the dimension-wise crossover with rotation-invariant differential operators, which we identify as a priority for future work.

## 14. Conclusions

This paper has proposed the Improved Coati Optimization Algorithm (ICOA), a multi-strategy enhanced version of the Coati Optimization Algorithm that addresses the original algorithm’s limitations in search diversity, exploration capability, and exploitation efficiency. Four complementary strategies were introduced: (1) Dynamic Adaptive Step-Size Optimization, combining Lévy flights with Student’s *t*-distribution perturbations; (2) Population-Adaptive Dynamic Perturbation incorporating differential evolution operators; (3) Iterative-Cyclic Population Differential Perturbation with sinusoidal scheduling; and (4) Cosine-Adaptive Gaussian Perturbation for refined exploitation. Each strategy is probabilistically activated at its respective decision point, maintaining behavioral diversity while enhancing search performance.

Comprehensive experimental evaluation on 51 benchmark functions across three CEC suites (CEC2017, CEC2020, CEC2022) demonstrated that ICOA consistently achieves overall rank 1 in Friedman test comparisons. On CEC2020, ICOA achieved a Friedman mean rank of 1.000 across all 10 functions, though the magnitude of advantage varies by function type. Scalability analysis across dimensions 10 through 100 confirmed that ICOA’s performance advantage is maintained and even strengthened at higher dimensionalities, with the Friedman mean rank increasing only marginally from 1.000 to 1.207 over a tenfold dimension increase.

The ablation study confirmed that each individual strategy provides measurable improvement over the baseline COA, with Strategy 3 (Iterative-Cyclic Differential Perturbation) contributing the most as a standalone enhancement. The pairwise combination analysis revealed that Strategies 1 and 3 exhibit the strongest synergy, consistent with their complementary phase targeting. The full ICOA significantly outperformed all single-strategy variants and all pairwise combinations, demonstrating that all four strategies contribute non-redundantly. A failure case analysis identified that ICOA’s dimension-wise crossover operator is suboptimal on rotated unimodal functions, where rotation-invariant algorithms such as ADE hold a structural advantage. An evaluation on six constrained engineering design problems further validated ICOA’s practical applicability, with statistically significant superiority on all test cases.

Future work will pursue four directions:

1. Adaptive strategy activation probability based on real-time population diversity and fitness improvement indicators.
2. Rotation-invariant operator design for Strategy 2’s crossover to address the identified weakness on rotated landscapes (Section 13.1).
3. Extension to multi-objective and discrete optimization problems with adaptive constraint handling.
4. Generalization of the probabilistic multi-strategy framework to other metaheuristic algorithms, such as GWO, WOA, and SSA.

## Figures and Tables

**Table 1:** ICOA-specific parameter settings.

Parameter	Value	Justification
Lévy stability index β	1.5	Standard choice [18]
*t*-distribution degrees of freedom ν	5	Moderate heavy tails [20]
Strategy activation probability	0.5	Equal blend of original and improved
Population size *N*	30	Standard for CEC benchmarks
Maximum iterations $T_{\max}$	1000	Standard for CEC benchmarks

**Table 2:** Parameter settings for comparison algorithms.

Algorithm	Key Parameters	Source
COA	No additional parameters	[11]
WAA	No additional parameters	[17]
SSOA	$P_{dp}=0.1$, $G_{c}=1.9$, $s_{f}=18$	[31]
NRBO	Δx=0.001	[32]
RFO	α=0.1, habitat range default	[33]
MSHOA	h=[5,0], M=5, $N_{\min}=5$	[34]
SABO	No additional parameters	[35]
VPPSO	$c_{1}=c_{2}=2.0$, w=[0.9,0.4]	[36]
ADE	F=0.5, CR=0.9, archive ratio =1	[25]
EIDBO	Default as in original	[15]
Clb_GWO	a=[2,0]	[7]
ASFSSA	ST=0.8, SD=0.2	[37]

**Table 3:** Friedman test rankings on CEC2017 benchmark functions (D=100).

Algorithm	Mean Rank	Overall Rank
**ICOA**	**1.207**	**1**
WAA	2.414	2
NRBO	3.414	3
SABO	3.517	4
RFO	5.517	5
MSHOA	5.552	6
COA	6.897	7
SSOA	7.483	8

**Table 4:** Mean and standard deviation on CEC2017 unimodal and multimodal functions (D=100). The best mean values are in **bold**.

Func.	Metric	ICOA	COA	WAA	SSOA	NRBO	RFO	MSHOA	SABO
F1	Mean	4.44E+9	2.72E+11	**4.79E+8**	2.68E+11	1.70E+11	2.78E+11	2.64E+11	1.20E+11
	Std	1.87E+9	9.44E+9	1.77E+8	6.33E+9	1.07E+10	3.09E+10	1.72E+10	1.51E+10
F3	Mean	**2.53E+5**	3.57E+5	4.22E+5	8.59E+5	3.41E+5	3.84E+5	3.72E+5	3.42E+5
	Std	2.04E+4	1.00E+4	1.89E+5	2.55E+6	4.30E+4	5.50E+4	3.08E+4	1.27E+4
F4	Mean	1.59E+3	1.08E+5	**1.09E+3**	1.04E+5	2.82E+4	9.41E+4	9.35E+4	2.11E+4
	Std	3.52E+2	1.35E+4	6.51E+1	8.15E+3	5.39E+3	1.64E+4	2.17E+4	5.53E+3
F5	Mean	**1.38E+3**	2.13E+3	1.44E+3	2.16E+3	2.01E+3	2.04E+3	2.09E+3	1.89E+3
	Std	7.31E+1	4.78E+1	4.21E+1	4.52E+1	6.93E+1	9.26E+1	7.35E+1	1.15E+2
F6	Mean	**6.62E+2**	7.12E+2	6.78E+2	7.18E+2	7.03E+2	7.03E+2	7.10E+2	7.04E+2
	Std	4.54E+0	3.96E+0	2.63E+0	3.92E+0	4.95E+0	6.58E+0	5.86E+0	5.69E+0
F7	Mean	**2.95E+3**	4.02E+3	3.68E+3	4.03E+3	3.68E+3	4.44E+3	4.00E+3	3.35E+3
	Std	2.25E+2	8.47E+1	5.76E+1	6.32E+1	1.85E+2	3.33E+2	9.85E+1	1.83E+2
F8	Mean	**1.78E+3**	2.60E+3	1.93E+3	2.64E+3	2.42E+3	2.49E+3	2.55E+3	2.31E+3
	Std	1.03E+2	4.00E+1	4.35E+1	4.84E+1	9.21E+1	1.03E+2	6.58E+1	9.13E+1
F9	Mean	**2.88E+4**	7.99E+4	5.66E+4	8.80E+4	6.99E+4	8.21E+4	7.95E+4	7.62E+4
	Std	1.74E+3	4.04E+3	5.20E+3	4.81E+3	5.66E+3	9.17E+3	4.19E+3	5.84E+3
F10	Mean	**1.73E+4**	3.30E+4	1.86E+4	3.35E+4	3.10E+4	3.03E+4	3.16E+4	3.21E+4
	Std	1.65E+3	8.12E+2	1.51E+3	8.66E+2	1.23E+3	1.40E+3	1.94E+3	6.77E+2

**Table 5:** Mean and standard deviation on CEC2017 hybrid and composition functions (D=100). The best mean values are in **bold**.

Func.	Metric	ICOA	COA	WAA	SSOA	NRBO	RFO	MSHOA	SABO
F11	Mean	**1.92E+4**	2.77E+5	1.52E+5	4.13E+6	1.12E+5	1.89E+5	2.02E+5	1.86E+5
	Std	6.10E+3	5.21E+4	3.30E+4	1.27E+7	2.29E+4	4.16E+4	2.68E+4	2.39E+4
F12	Mean	**2.60E+8**	2.09E+11	4.46E+8	1.99E+11	6.76E+10	1.78E+11	1.76E+11	4.42E+10
	Std	1.03E+8	2.06E+10	1.04E+8	1.41E+10	1.77E+10	3.46E+10	3.20E+10	1.49E+10
F13	Mean	**1.44E+5**	4.90E+10	2.28E+6	4.62E+10	1.47E+10	3.86E+10	4.27E+10	6.52E+9
	Std	1.27E+5	5.01E+9	4.28E+5	4.54E+9	4.52E+9	8.97E+9	1.04E+10	3.79E+9
F14	Mean	**6.42E+5**	9.46E+7	1.90E+6	1.94E+8	2.20E+7	3.40E+7	7.29E+7	2.07E+7
	Std	3.57E+5	3.76E+7	6.29E+5	6.42E+7	1.09E+7	2.74E+7	5.05E+7	6.77E+6
F15	Mean	**1.32E+4**	2.62E+10	5.87E+5	2.41E+10	4.34E+9	1.45E+10	2.04E+10	1.10E+9
	Std	6.68E+3	3.75E+9	1.27E+5	4.03E+9	2.03E+9	5.45E+9	8.16E+9	9.81E+8
F16	Mean	**6.68E+3**	2.61E+4	8.52E+3	2.51E+4	1.42E+4	1.80E+4	2.08E+4	1.38E+4
	Std	7.02E+2	3.10E+3	8.66E+2	2.50E+3	1.25E+3	2.34E+3	3.40E+3	1.40E+3
F17	Mean	6.18E+3	1.52E+7	**6.12E+3**	1.22E+7	5.83E+4	1.61E+6	1.16E+7	3.02E+4
	Std	6.27E+2	1.31E+7	5.81E+2	7.94E+6	7.30E+4	1.74E+6	1.49E+7	2.46E+4
F18	Mean	**8.16E+5**	2.81E+8	3.72E+6	3.41E+8	3.34E+7	5.68E+7	2.02E+8	2.11E+7
	Std	4.39E+5	1.36E+8	2.01E+6	1.12E+8	1.50E+7	5.46E+7	2.02E+8	8.50E+6
F19	Mean	**1.92E+4**	2.52E+10	3.77E+6	2.63E+10	3.72E+9	1.65E+10	2.06E+10	2.10E+9
	Std	2.20E+4	4.45E+9	2.13E+6	3.26E+9	1.58E+9	4.64E+9	8.56E+9	1.92E+9
F20	Mean	**5.66E+3**	7.87E+3	6.59E+3	8.58E+3	7.06E+3	6.83E+3	7.63E+3	7.78E+3
	Std	5.62E+2	4.21E+2	4.95E+2	3.46E+2	4.54E+2	5.12E+2	4.90E+2	2.41E+2
F21	Mean	**3.41E+3**	4.95E+3	4.50E+3	5.23E+3	4.09E+3	4.87E+3	4.47E+3	4.51E+3
	Std	1.70E+2	2.25E+2	1.62E+2	2.72E+2	1.43E+2	2.23E+2	1.78E+2	2.32E+2
F22	Mean	2.22E+4	3.53E+4	**2.13E+4**	3.62E+4	3.29E+4	3.32E+4	3.43E+4	3.45E+4
	Std	1.12E+3	7.39E+2	1.26E+3	8.12E+2	1.53E+3	1.66E+3	1.24E+3	8.14E+2
F23	Mean	**3.90E+3**	6.74E+3	6.63E+3	8.53E+3	4.96E+3	7.83E+3	6.00E+3	5.50E+3
	Std	1.71E+2	3.01E+2	8.42E+2	4.86E+2	2.77E+2	6.94E+2	3.98E+2	2.93E+2
F24	Mean	**4.49E+3**	1.04E+4	1.04E+4	1.38E+4	6.04E+3	1.21E+4	9.35E+3	7.21E+3
	Std	1.78E+2	8.54E+2	2.31E+3	8.01E+2	3.42E+2	1.22E+3	1.22E+3	3.60E+2
F25	Mean	4.25E+3	2.98E+4	**3.72E+3**	2.85E+4	1.50E+4	3.01E+4	2.90E+4	1.23E+4
	Std	3.01E+2	2.03E+3	5.10E+1	2.09E+3	1.60E+3	4.10E+3	3.26E+3	1.91E+3
F26	Mean	**2.24E+4**	5.32E+4	2.74E+4	5.70E+4	3.69E+4	5.23E+4	5.56E+4	3.87E+4
	Std	2.01E+3	2.05E+3	1.28E+3	2.55E+3	3.06E+3	4.94E+3	4.23E+3	2.53E+3
F27	Mean	**3.99E+3**	1.51E+4	7.75E+3	1.60E+4	6.05E+3	1.27E+4	1.09E+4	6.55E+3
	Std	1.65E+2	1.92E+3	2.15E+3	1.37E+3	5.78E+2	1.88E+3	1.31E+3	1.04E+3
F28	Mean	4.59E+3	3.06E+4	**3.87E+3**	3.59E+4	1.98E+4	3.51E+4	3.55E+4	1.62E+4
	Std	4.39E+2	1.17E+3	6.22E+1	1.87E+3	1.63E+3	4.07E+3	4.79E+3	1.99E+3
F29	Mean	**7.99E+3**	8.60E+5	1.07E+4	7.66E+5	2.41E+4	1.50E+5	1.36E+6	2.15E+4
	Std	8.37E+2	6.67E+5	8.34E+2	3.08E+5	8.90E+3	1.55E+5	1.95E+6	8.10E+3
F30	Mean	**1.83E+6**	4.43E+10	4.98E+7	4.25E+10	1.04E+10	3.36E+10	3.81E+10	6.09E+9
	Std	1.90E+6	5.12E+9	1.27E+7	6.36E+9	4.11E+9	8.19E+9	1.19E+10	3.68E+9

**Table 6:** Mean and standard deviation of fitness values on CEC2020 benchmark functions (D=10). The best mean values are highlighted in **bold**.

Func.	Metric	ICOA	COA	WAA	SSOA	NRBO	RFO	MSHOA	SABO
F1	Mean	**2.08E+3**	9.15E+9	4.53E+4	1.08E+10	4.80E+8	6.22E+8	5.17E+9	1.81E+8
	Std	2.57E+3	3.81E+9	1.69E+4	2.92E+9	2.87E+8	7.07E+8	5.28E+9	1.69E+8
F2	Mean	**1.82E+3**	2.57E+3	2.25E+3	3.34E+3	2.24E+3	1.96E+3	2.47E+3	2.77E+3
	Std	2.75E+2	1.57E+2	1.95E+2	2.35E+2	1.92E+2	2.70E+2	3.61E+2	1.88E+2
F3	Mean	**7.45E+2**	7.99E+2	8.31E+2	8.30E+2	7.67E+2	7.55E+2	8.13E+2	7.61E+2
	Std	1.24E+1	2.13E+1	1.35E+1	1.08E+1	1.84E+1	2.22E+1	2.20E+1	1.32E+1
F4	Mean	**1.90E+3**	3.12E+4	1.91E+3	2.21E+5	1.93E+3	2.21E+3	1.72E+5	2.25E+3
	Std	8.28E−01	3.35E+4	2.79E+0	1.21E+5	6.06E+1	9.42E+2	2.83E+5	1.25E+3
F5	Mean	**1.94E+3**	3.81E+5	1.33E+5	6.07E+5	6.50E+3	2.96E+3	5.23E+5	7.45E+3
	Std	1.24E+2	1.88E+5	5.61E+4	1.35E+5	4.30E+3	3.20E+3	3.39E+5	1.44E+4
F6	Mean	**1.64E+3**	2.03E+3	2.09E+3	2.49E+3	1.80E+3	1.75E+3	1.99E+3	1.94E+3
	Std	5.15E+1	1.85E+2	2.14E+2	2.19E+2	1.01E+2	1.08E+2	1.66E+2	1.29E+2
F7	Mean	**2.19E+3**	1.51E+4	8.59E+3	1.41E+6	4.76E+3	2.41E+3	1.57E+4	1.11E+4
	Std	6.24E+1	1.13E+4	7.07E+3	3.44E+6	5.25E+3	2.64E+2	1.11E+4	1.02E+4
F8	Mean	**2.30E+3**	3.08E+3	2.70E+3	3.15E+3	2.35E+3	2.40E+3	2.70E+3	2.34E+3
	Std	2.18E+1	3.72E+2	7.79E+2	2.98E+2	3.21E+1	1.10E+2	2.96E+2	6.78E+1
F9	Mean	**2.52E+3**	2.85E+3	2.89E+3	2.97E+3	2.78E+3	2.76E+3	2.76E+3	2.77E+3
	Std	5.15E+1	7.92E+1	1.26E+2	5.58E+1	4.95E+1	6.83E+1	8.16E+1	9.88E+0
F10	Mean	**2.91E+3**	3.45E+3	2.96E+3	3.38E+3	2.96E+3	2.97E+3	3.32E+3	2.95E+3
	Std	1.90E+1	2.33E+2	3.91E+1	1.75E+2	3.63E+1	4.52E+1	2.60E+2	2.66E+1

**Table 7:** Friedman test rankings on CEC2020 benchmark functions (D=10).

Algorithm	Mean Rank	Overall Rank
**ICOA**	**1.000**	**1**
RFO	3.000	2
NRBO	3.500	3
SABO	3.900	4
WAA	4.700	5
MSHOA	5.800	6
COA	6.300	7
SSOA	7.800	8

**Table 8:** Mean and standard deviation of fitness values on CEC2022 benchmark functions (D=10). The best mean values are highlighted in **bold**.

Func.	Metric	ICOA	COA	VPPSO	ADE	EIDBO	RLQFAO	Clb_GWO	ASFSSA
F1	Mean	3.00E+2	7.87E+3	3.03E+2	2.30E+3	8.23E+2	**3.00E+2**	4.97E+2	3.00E+2
	Std	5.71E−02	1.69E+3	1.23E+1	1.06E+3	1.26E+3	1.32E−03	2.17E+2	4.04E−07
F2	Mean	**4.01E+2**	1.69E+3	4.07E+2	4.08E+2	4.48E+2	4.01E+2	4.15E+2	4.14E+2
	Std	3.30E+0	7.08E+2	1.28E+1	7.39E−01	3.83E+1	2.79E+0	1.79E+1	2.12E+1
F3	Mean	6.03E+2	6.48E+2	6.09E+2	**6.00E+2**	6.09E+2	6.02E+2	6.06E+2	6.03E+2
	Std	1.75E+0	9.29E+0	8.65E+0	2.89E−13	8.88E+0	3.16E+0	3.69E+0	3.96E+0
F4	Mean	8.20E+2	8.54E+2	8.23E+2	**8.19E+2**	8.26E+2	8.21E+2	8.20E+2	8.32E+2
	Std	5.66E+0	8.86E+0	7.07E+0	5.12E+0	1.09E+1	6.80E+0	9.02E+0	5.93E+0
F5	Mean	9.20E+2	1.43E+3	9.32E+2	**9.00E+2**	9.70E+2	9.31E+2	9.25E+2	1.35E+3
	Std	3.42E+1	1.80E+2	3.75E+1	1.52E−02	6.43E+1	3.43E+1	1.80E+1	2.06E+2
F6	Mean	**1.83E+3**	1.18E+7	4.38E+3	4.89E+3	5.75E+3	3.31E+3	6.01E+3	5.30E+3
	Std	1.43E+1	1.60E+7	2.00E+3	2.34E+3	2.27E+3	1.38E+3	1.79E+4	2.31E+3
F7	Mean	2.03E+3	2.09E+3	2.05E+3	**2.01E+3**	2.04E+3	2.02E+3	2.03E+3	2.03E+3
	Std	6.58E+0	1.73E+1	1.48E+1	8.74E+0	2.32E+1	9.67E+0	7.40E+0	2.78E+1
F8	Mean	2.22E+3	2.24E+3	2.23E+3	**2.22E+3**	2.23E+3	2.22E+3	2.22E+3	2.22E+3
	Std	4.61E+0	6.45E+0	2.40E+0	3.59E+0	8.75E+0	8.33E−01	7.13E+0	1.60E+0
F9	Mean	**2.53E+3**	2.74E+3	2.54E+3	2.53E+3	2.58E+3	2.53E+3	2.53E+3	2.54E+3
	Std	2.53E−13	4.58E+1	2.69E+1	0.00E+0	5.61E+1	5.54E−07	6.60E+0	3.73E+1
F10	Mean	2.52E+3	2.73E+3	2.56E+3	**2.49E+3**	2.54E+3	2.50E+3	2.50E+3	2.54E+3
	Std	4.40E+1	1.41E+2	5.96E+1	4.50E+1	6.37E+1	1.44E−01	2.79E−01	6.11E+1
F11	Mean	**2.60E+3**	3.88E+3	2.80E+3	2.88E+3	2.71E+3	2.63E+3	2.71E+3	2.72E+3
	Std	2.48E+1	4.60E+2	1.43E+2	5.51E+1	1.13E+2	6.13E+1	4.53E+1	1.40E+2
F12	Mean	**2.86E+3**	2.95E+3	2.86E+3	2.86E+3	2.87E+3	2.87E+3	2.86E+3	2.87E+3
	Std	1.80E+0	4.72E+1	1.26E+0	1.65E+0	1.08E+1	5.16E+0	1.98E+0	9.84E+0

**Table 9:** Friedman test rankings on CEC2022 benchmark functions (D=10).

Algorithm	Mean Rank	Overall Rank
**ICOA**	**2.208**	**1**
ADE	2.542	2
RLQFAO	2.750	3
Clb_GWO	4.333	4
ASFSSA	4.833	5
VPPSO	5.167	6
EIDBO	6.167	7
COA	8.000	8

**Table 10:** Friedman mean ranks across different dimensions on CEC2017.

Algorithm	10D	30D	50D	100D
**ICOA**	**1.000**	**1.034**	**1.138**	**1.207**
WAA	5.310	3.345	2.655	2.414
NRBO	3.345	3.379	3.448	3.414
SABO	4.172	3.517	3.517	3.517
RFO	2.759	4.138	4.759	5.517
MSHOA	5.483	5.931	5.931	5.552
COA	6.138	6.862	7.000	6.897
SSOA	7.793	7.793	7.552	7.483

**Table 11:** Friedman test rankings for the ablation study on CEC2017 (D=10).

Variant	Mean Rank	Overall Rank
**ICOA (all strategies)**	**1.103**	**1**
COA3 (Strategy 3 only)	2.103	2
COA1 (Strategy 1 only)	2.793	3
SABO (reference)	3.966	4
COA2 (Strategy 2 only)	4.379	5
COA4 (Strategy 4 only)	5.000	6
COA (baseline)	5.621	7
MSHOA (reference)	6.621	8

**Table 12:** Friedman mean ranks for different strategy activation probabilities on CEC2017 (D=10).

Activation Probability	Friedman Mean Rank	Overall Rank
p=0.3	2.103	2
p=0.5	**1.207**	**1**
p=0.7	2.690	3

**Table 13:** Friedman mean ranks for pairwise strategy combinations on CEC2017 (D=10). S*i* + S*j* denotes the variant incorporating only Strategies *i* and *j*.

Combination	Friedman Mean Rank	Rank
**ICOA (S1 + S2 + S3 + S4)**	**1.103**	**1**
S1 + S3	1.552	2
S1 + S2	2.138	3
S3 + S4	2.241	4
S2 + S3	2.310	5
S1 + S4	2.793	6
S2 + S4	3.379	7
COA (baseline)	5.621	8

**Table 14:** Characteristics of the six engineering design problems. *D*: number of design variables; *g*: number of inequality constraints.

ID	Problem Name	*D*	*g*	Objective
P1	Speed Reducer Weight Minimization	7	11	Minimize gear reducer weight
P2	Industrial Refrigeration System Design	14	15	Minimize total system cost
P3	Multiple Disk Clutch Brake Design	5	7	Minimize brake material weight
P4	Ten-Bar Truss Design	10	3	Minimize structural weight
P5	Rolling Element Bearing Design	10	9	Minimize dynamic load rating
P6	Gear Train Design	4	0	Minimize gear ratio error

**Table 15:** Results on six constrained engineering design problems. Problem IDs correspond to Table 14. The best average values are highlighted in **bold**.

Prob.	Metric	ICOA	COA	SSOA	SABO	NRBO	WAA	MSHOA	RFO
P1	Best	2.99E+3	3.02E+3	3.76E+3	3.04E+3	3.00E+3	3.01E+3	3.04E+3	2.99E+3
	Mean	**3.00E+3**	1.04E+13	1.41E+14	4.29E+3	3.05E+3	3.02E+3	3.16E+3	3.00E+3
	Std	9.96E+0	3.00E+13	1.11E+14	7.28E+2	4.98E+1	8.10E+0	7.85E+1	1.01E+1
P2	Best	3.22E−02	1.27E+13	1.12E+16	7.21E−01	4.48E−02	7.40E−01	1.22E+3	3.96E+1
	Mean	**1.35E−01**	3.94E+16	1.98E+17	1.38E+16	6.24E+13	7.20E+14	2.85E+15	1.91E+15
	Std	3.33E−01	2.23E+16	2.05E+17	2.06E+16	2.38E+14	4.04E+14	3.59E+15	2.44E+15
P3	Best	4.67E−02	5.14E−02	3.31E−01	2.36E−01	2.35E−01	2.35E−01	2.38E−01	2.35E−01
	Mean	**1.78E−01**	2.56E−01	3.31E−01	2.73E−01	2.35E−01	2.35E−01	2.56E−01	2.35E−01
	Std	8.36E−02	8.02E−02	0.00E+0	2.30E−02	4.32E−07	2.22E−06	9.80E−03	3.84E−13
P4	Best	5.25E+2	6.12E+2	7.18E+2	5.55E+2	5.36E+2	6.25E+2	5.45E+2	5.27E+2
	Mean	**5.30E+2**	7.00E+2	8.61E+2	5.94E+2	5.60E+2	7.16E+2	5.91E+2	5.59E+2
	Std	2.98E+0	5.01E+1	9.21E+1	2.79E+1	1.32E+1	4.47E+1	1.85E+1	2.76E+1
P5	Best	1.70E+4	1.71E+4	3.52E+4	1.76E+4	1.70E+4	1.70E+4	1.95E+4	1.70E+4
	Mean	**1.70E+4**	2.05E+14	1.25E+16	2.44E+4	1.71E+4	1.80E+4	2.37E+4	1.77E+4
	Std	3.07E+1	1.12E+15	1.13E+16	4.83E+3	1.21E+2	3.07E+3	3.44E+3	1.03E+3
P6	Best	0.00E+0	0.00E+0	7.00E−08	1.73E−14	0.00E+0	3.52E−16	2.45E−11	0.00E+0
	Mean	**0.00E+0**	4.63E−12	1.99E−03	3.04E−12	1.76E−31	3.99E−13	3.94E−08	1.30E−32
	Std	0.00E+0	2.10E−11	5.58E−03	4.55E−12	9.45E−31	9.24E−13	6.00E−08	1.87E−32

## Data Availability

The MATLAB source code for ICOA, all comparison algorithm implementations, benchmark function definitions, constraint handling routines, and experimental scripts are provided as Supplementary Materials accompanying this submission. The complete code repository will be publicly released on GitHub upon the acceptance of this article; during the review process, all materials were available from the corresponding author upon request. The reproducibility protocol in Section 5.5 specifies the random seeds, runtime environment, and termination criteria required to reproduce every numerical result reported herein.

## Supplementary Materials

The following supporting information can be downloaded at: https://www.mdpi.com/article/10.3390/biomimetics11040254/s1.

## Abbreviations

The following abbreviations are used in this manuscript:

ICOA	Improved Coati Optimization Algorithm
COA	Coati Optimization Algorithm
CEC	Congress on Evolutionary Computation
DE	Differential Evolution
PSO	Particle Swarm Optimization
GA	Genetic Algorithm
GWO	Grey Wolf Optimizer
WOA	Whale Optimization Algorithm
HHO	Harris Hawks Optimization
SSA	Salp Swarm Algorithm

## Appendix A. Wilcoxon Rank-Sum Test p-Values

Table A1, Table A2 and Table A3 present the Wilcoxon rank-sum test *p*-values for pairwise comparisons between ICOA and each competitor. Values below the Bonferroni-adjusted significance threshold are marked with “≈” (no significant difference). The win/tie/loss (W/T/L) summary is provided for each competitor.

**Table A1:** Wilcoxon rank-sum test *p*-values: ICOA vs. each competitor on CEC2017 (D=100). “≈”: no significant difference.

	COA	WAA	SSOA	NRBO	RFO	MSHOA	SABO
W/T/L	29/0/0	28/1/0	29/0/0	29/0/0	29/0/0	29/0/0	29/0/0
*All 29 functions yield $p<3.02\times 10^{-11}$ against COA, SSOA, NRBO, RFO, MSHOA, and SABO.*							
*Against WAA: 28 functions with p<0.05; F17: p=0.935 (≈).*							

**Table A2:** Wilcoxon rank-sum test summary: ICOA vs. each competitor on CEC2020 (D=10). “≈”: no significant difference.

	COA	WAA	SSOA	NRBO	RFO	MSHOA	SABO
W/T/L	10/0/0	10/0/0	10/0/0	10/0/0	8/2/0	10/0/0	10/0/0
*Against RFO: F2 (p=0.088, ≈), F3 (p=0.070, ≈); remaining 8 functions p<0.05.*							
*Against all other competitors: all 10 functions p<0.05.*							

**Table A3:** Wilcoxon rank-sum test summary: ICOA vs. each competitor on CEC2022 (D=10). W: ICOA significantly better (p<0.05); T: no significant difference (p≥0.05); L: ICOA significantly worse (p<0.05 in favor of competitor).

	COA	VPPSO	ADE	EIDBO	RLQFAO	Clb_GWO	ASFSSA
W/T/L	12/0/0	8/2/2	5/3/4	9/2/1	7/3/2	7/3/2	9/2/1
*Against COA:*	*all 12 functions $p<3.02\times 10^{-11}$.*						
*Against ADE:*	*ADE is significantly better on F3 (p=0.007) and F5 (p=0.003), where it achieves*						
	*near-optimal values with near-zero Std. On F7 and F8, the differences are not statistically*						
	*significant (p>0.05), with absolute margins below 1%. ICOA dominates on*						
	*multimodal/composition functions (F6, F9, F11, F12).*						
